# Evaluation of organ visualisation and biopsy quality during left-sided coelioscopy in rescued Northern boobooks (*Ninox japonica*)

**DOI:** 10.17221/9/2026-VETMED

**Published:** 2026-09-30

**Authors:** Jang-Hee Han, So-Eun Ryu, Hyeok-Won An, Hyeok Jin Son, Seung Yoon Ahn, Jinyoung Kim, Jun Won Park, Seong-Chan Yeon

**Affiliations:** ^1^Department of Veterinary Clinical Sciences, College of Veterinary Medicine and Research Institute for Veterinary Science, Seoul National University, Seoul, Republic of Korea; ^2^Seoul Wildlife Centre, Seoul National University, Seoul, Republic of Korea; ^3^Department of Laboratory Animal Medicine, College of Veterinary Medicine, Seoul National University, Seoul, Republic of Korea

**Keywords:** avian histopathology, coelioscopic-guided biopsy, minimally invasive technique, raptor medicine, Strigiformes

## Abstract

Intra-vital diagnosis in wild raptors can be confounded by their stoic nature, often leading to cryptic morbidity. While coelioscopy offers a minimally invasive alternative, its performance regarding diagnostic utility and biopsy quality remains uncharacterised in small owls. Eight Northern boobooks (*Ninox japonica*) underwent diagnostic coelioscopy using a 2.7 mm rigid endoscope. Ease of coelomic entry was scored on a predefined scale, followed by a clockwise systematic survey of the anterior, dorsal, and caudal quadrants, during which organ visibility was graded. Lung, liver, and kidney biopsies were acquired; for four euthanised owls, these were compared with necropsy tissues to assess diagnostic concordance. Two-thirds of the biopsy specimens were considered identifiable as the target organ based on characteristic histological features. Kidney samples were of excellent diagnostic quality, and liver samples were of fair quality, with both demonstrating high consistency with post-mortem findings. In contrast, lung biopsies were frequently compromised by severe iatrogenic artefacts, such as parenchymal collapse and fragmentation, rendering their histopathological value poor. Coelioscopy affords reliable intra-vital access to liver and kidney tissue in Northern boobooks, supporting clinical evaluation of hepatorenal disease. Given the frequent artefact burden, pulmonary specimens should be interpreted cautiously and supplemented by alternative sampling strategies.

Diseased wild raptors tend to respond stoically to their illness, obscuring early detection of underlying health issues until the disease has worsened to irreversible states such as organ failure (Heatley et al. 2001; Black et al. 2011). Clinical signs, radiography, and clinical pathology have hitherto been mainstays of intra-vital diagnosis in wildlife practice; however, relying solely on these techniques is increasingly outdated, as they are diagnostically inconclusive (Divers 2010; Desmarchelier and Ferrell 2015).

Such uncertainty often constrains clinicians to symptomatic care, precluding aetiology-directed treatment and prognostication (Divers 2010). Consequently, manageable conditions may be diagnosed only at necropsy, amounting to a retrospective diagnosis. Therefore, securing a definitive diagnosis during life is crucial for improving clinical decision-making and outcomes.

In this context, diagnostic coelioscopy is widely acknowledged in avian practice as an advanced modality for direct coelomic assessment and necessary intervention. A rigid endoscopy system enables macroscopic visceral inspection and controlled instrument access to targeted lesions through a minimal incision (Seok et al. 2016). This technique has been utilised across avian species, particularly for the evaluation and biopsy of major parenchymal organs, such as liver, kidneys, and spleen, as well as the bursa of Fabricius (Muller et al. 2004; Hernandez-Divers et al. 2006; Seok et al. 2016).

Tissue collection via biopsy performed during this procedure represents an effective tool for uncovering the aetiology in many clinical cases, offering crucial histopathological evidence in the living patient, particularly in wildlife species for which reference data are scarce (Muller et al. 2004; Mejia-Fava et al. 2013; Divers 2015). Despite these clinical benefits, systematic studies evaluating the diagnostic yield and procedural safety in small owls remain undefined.

Therefore, we selected the Northern boobook (*Ninox japonica*), a small-bodied strigid owl frequently admitted to wildlife rescue centres in Korea, as a model species for this study. Because rescued individuals present diverse clinical profiles, this species was an ideal candidate for evaluating the feasibility and diagnostic utility of coelioscopy under conditions that reflect actual clinical practice. Accordingly, we assessed the procedure’s visual diagnostic capability by quantitatively evaluating the confirmation and accessibility of major organs via coelioscopy. In addition, we examined whether the minimally invasive biopsy samples possessed sufficient diagnostic value.

## MATERIAL AND METHODS

### Experimental animals

This study focused on the Northern boobooks admitted to a regional wildlife rescue centre in Korea between May and August 2025. The age and sex of these individuals were undetermined upon admission, owing to their wild origin and sexually monomorphic plumages. All owls underwent standard triage and were hospitalised for clinical intervention. During the holding period, they were provided with thawed one-day-old chicks and had *ad* *libitum* access to fresh water to ensure adequate nutrition and hydration. Each received a comprehensive diagnostic workup comprising physical examination, orthogonal radiography, blood analysis, and ophthalmic examination. Based on this initial workup, individuals unsuitable for coelioscopy by virtue of marked coelomic fat deposits indicative of obesity, ascites, or suspected haemostatic compromise were excluded from the study. Ascites was diagnosed from dyspnoea and coelomic distension on physical examination, together with a compressed air sac silhouette and an overall ground-glass appearance of the coelomic cavity on radiography. Haemostatic compromise was suspected from clinical signs such as subcutaneous ecchymoses or prolonged bleeding from wounds or venepuncture sites. A total of eight owls, all of which had been admitted due to presumed trauma resulting from collisions with man-made structures, were selected for the final study population. The owls were deemed non-releasable due to a lack of significant clinical improvement despite intensive veterinary care. Given their poor prognoses, coelioscopy was undertaken as a final diagnostic step to avoid futile therapeutic interventions prior to humane euthanasia. This study was approved by the Institutional Animal Care and Use Committee of Seoul National University (Approval No. SNU-250219-3). In addition, all animal-related procedures were conducted lawfully at a nationally accredited facility with permission from the Administrator of the Korea Heritage Service, as stipulated in the Act on the Preservation and Utilisation of Natural Heritage.

### Anaesthesia

Food was withheld for 6 h to 12 h before induction of general anaesthesia for coelioscopy and subsequent euthanasia. Meloxicam (Metacam^®^; Boehringer Ingelheim, Seoul, Republic of Korea) was administered intramuscularly at 0.5 mg/kg for preemptive analgesia. Induction was performed using an inhalation anaesthesia machine (Multiplus-MEVD; Royal Medical Co. Ltd., Bucheon, Republic of Korea) by delivering 5% isoflurane (Isotroy 100^®^; Troikaa Pharmaceuticals Ltd., Ahmedabad, India) in 100% oxygen at a flow rate of 2 l/min via a T-piece non-rebreathing circuit and a facemask fitted over the head. Once an adequate depth of anaesthesia was confirmed by loss of corneal reflex and pedal withdrawal reflexes, anaesthesia was maintained with 2−3% isoflurane under spontaneous ventilation. The airway was secured with a 2.0 mm uncuffed endotracheal tube, and intermittent positive pressure ventilation was provided using a pressure-limited mechanical ventilator (SAV03; Vetronic Services Ltd., Devon, UK) when spontaneous ventilation became insufficient. Basic physiological variables, including heart rate, respiratory rate, and body temperature, were monitored intraoperatively using a multiparameter monitor (GE Carescape B650; GE Healthcare, Chicago, IL, USA). To minimise the risk of intraoperative hypothermia, body temperature was maintained using a heating pad and an infrared heat lamp.

### Diagnostic coelioscopy and biopsy

The coelioscopic procedure was adapted from the single-portal technique described by Hernandez-Divers et al. (2006). Anaesthetised owls were placed in right lateral recumbency to expose the left flank. Both wings were secured dorsally, and the left hindlimb was retracted cranially and taped in place alongside the neck to maintain the position. Feathers over the intended entry site were plucked, and the surrounding plumage was reflected away from the surgical field and secured with adhesive tape. The surgical site was aseptically prepared with 70% alcohol and 2% chlorhexidine, then covered with a sterile transparent drape (SHS-5050V; KM Healthcare, Guri, Republic of Korea). A 5-mm skin incision was made with a No. 11 scalpel blade at a site caudal to the last rib, cranial to the femur, and ventral to the flexor cruris medialis muscle. Subsequently, blunt dissection through the body wall was performed using small mosquito forceps to establish a portal into the caudal thoracic air sac.

An endoscopy system was configured for diagnostic coelioscopy, comprising a high-definition camera system (1188 HD; Stryker, Kalamazoo, MI, USA) and a xenon light source (X8000; Stryker, Kalamazoo, MI, USA). To achieve visualisation and instrument passage, a 2.7-mm, 30° rigid telescope (J2330X; Shenda Endoscope Co. Ltd., Shenyang, P.R. China) housed within a 14-Fr operating sheath (N4014; Shenda Endoscope Co. Ltd., Shenyang, P.R. China) was used.

For biopsy procedures, a single-channel endoscope bridge (N4201X; Shenda Endoscope Co. Ltd., Shenyang, P.R. China) was mounted on the telescope to enable targeted tissue sampling through the working channel using 5-Fr biopsy forceps (N5151; Shenda Endoscope Co. Ltd., Shenyang, P.R. China) with oval, spoon-shaped jaws. Table 1 lists equipment specifications, and Figure 1 presents a schematic overview of the coelioscopic setup used in the Northern boobooks.

**Table 1:** Endoscopic equipment and instrumentation for diagnostic coelioscopy and coelioscopic biopsy in Northern boobooks (*Ninox japonica*)

System category	Components	Model (manufacturer)	Specifications
Imaging system	camera system	1188 HD (Stryker)	high-definition video with 1 280 × 1 024 output
	xenon light source	X8000 (Stryker)	300-watt xenon
			
Endoscope	telescope	J2330X (Shenda Endoscope)	30° rigid endoscope, 2.7 mm × 190 mm
	operating sheath	N4014 (Shenda Endoscope)	14 Fr × 135 mm
			
Biopsy instrumentation	working channel	N4201X (Shenda Endoscope)	Single channel endoscopic bridge
	Biopsy forceps	N5151 (Shenda Endoscope)	flexible type, oval spoon-shaped jaws, 5 Fr × 280 mm

**Figure 1:**
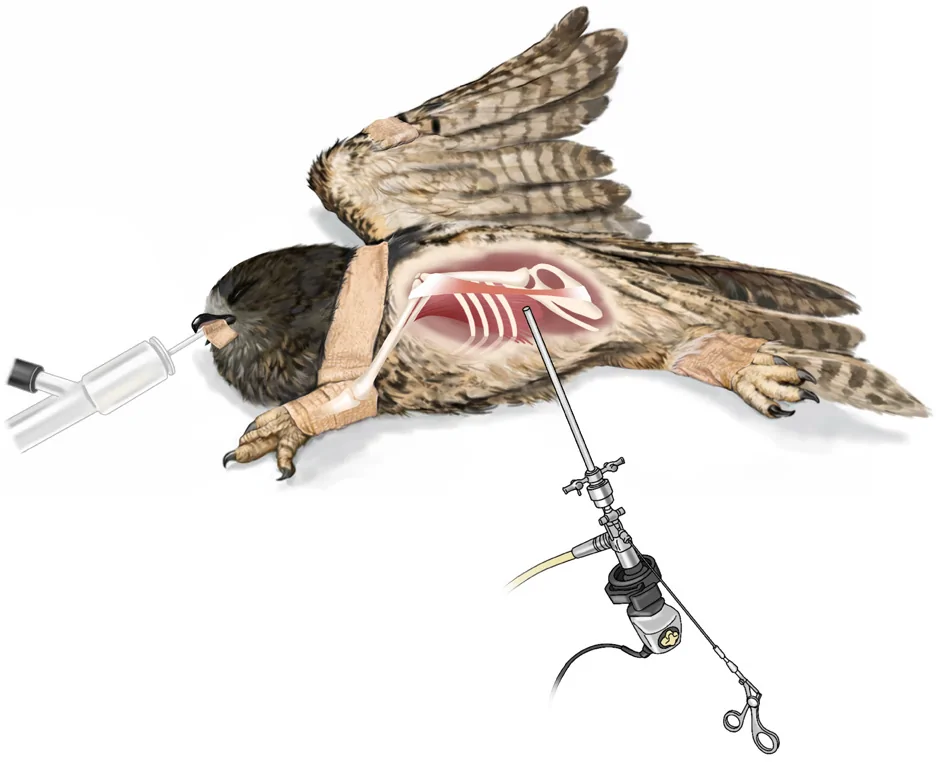
Schematic illustration of single-portal coelioscopy in Northern boobooks (*Ninox japonica*) via left caudal thoracic air sac The bird is positioned to expose the left flank, and a sheathed telescope is introduced at a site caudal to the last rib, cranial to the femur, and ventral to the flexor cruris medialis muscle. Adhesive tapes and transparent surgical drape have been omitted for visual clarity

The sheathed telescope was introduced through the created portal for visceral exploration. To ensure safe advancement, transillumination of the air sac membranes was utilised to delineate underlying viscera and vasculature, and the air sac membrane was then gently perforated with the tip of the operating sheath. Visceral evaluation was conducted by following a clockwise pattern, beginning with cranial structures and continuing towards the dorsal and caudal regions. When a more extensive exploration was required, the telescope was advanced into the cranial thoracic and abdominal air sacs to facilitate organ visualisation. The coelomic entry score (CES) was graded by the endoscopist on a previously established 5-point ordinal scale (Hernandez-Divers et al. 2004; Seok et al. 2016; Frei et al. 2020), with higher scores indicating faster and technically less demanding entry to the coelom: 1 = inaccessible (>15 min); 2 = poor (11–15 min); 3 = fair (6–10 min); 4 = good (2–5 min); and 5 = excellent (<2 min). Similarly, the endoscopic visibility score (EVS) for visceral organs (heart, lung, oesophagus, proventriculus, ventriculus, liver, gall bladder, spleen, pancreas, small and large intestines, cloaca, kidney, ureter, adrenal gland, and gonad) was graded on a separate 5-point scale adapted from the aforementioned literature. Lower scores denoted poor visualisation despite extensive exploration, whereas higher scores denoted clear identification with minimal manipulation, defined as follows: 1 = very poor (barely discernible or not visualised, even after prolonged inspection); 2 = poor (requiring substantial searching and manipulation); 3 = fair (requiring moderate searching and manipulation); 4 = good (readily locatable with slight manipulation); and 5 = excellent (immediate, clear visualisation with little visceral manipulation).

Following the diagnostic survey, coelioscopy-guided biopsies of the lung, liver, and kidney were obtained by advancing biopsy forceps through the working channel under direct visualisation. During pulmonary and hepatic sampling, the telescope was kept within the left caudal thoracic air sac. For the lung, it was oriented craniodorsally to visualise the peripheral lung parenchyma, and small fragments were gently grasped and retrieved while avoiding major vascular structures. To obtain hepatic samples, the telescope was redirected caudoventrally towards the hepatic lobe, and biopsy specimens were collected from its caudal margin. Renal biopsies were performed by advancing the telescope caudally into the abdominal air sac to expose the cranial division of the kidney, from which small parenchymal samples were collected. In each owl, one or two biopsy fragments were obtained per organ. After all biopsies had been collected, the coelomic cavity was re-inspected for active haemorrhage, and all endoscopic instruments were withdrawn. Upon conclusion of all procedures, euthanasia was performed via intravenous administration of T61 (MSD Animal Health, Seoul, Republic of Korea) at 0.5 ml/kg.

### Post-mortem examination and histopathology

To ensure that organ conditions at biopsy and necropsy were compared on an equivalent basis, a standard avian necropsy was performed on all individuals immediately following euthanasia. After the organs had been exposed, wedge samples were harvested with meticulous care taken to minimise tissue contamination and any artefactual change. All samples obtained from both biopsy and necropsy were immediately transferred to pre-labelled specimen containers filled with 10% neutral buffered formalin for histopathology. However, owing to budgetary constraints, samples from only four randomly selected individuals underwent histopathological examination. These were processed using a standard method: paraffin-embedded, sectioned at 3 µm, and stained with haematoxylin and eosin.

Histological slides were reviewed for morphological integrity, specifically preservation of tissue architecture and cellular detail, using a tiered assessment system. First, target organ confirmation was graded as definitive, probable, or unidentifiable, depending on whether histological features were sufficient to confirm tissue origin. Second, diagnostic quality was rated as good, limited, or poor, based on the retention of organ-specific structures and the severity of crush artefacts. Third, these parameters were integrated to determine overall diagnostic adequacy, categorised as adequate, suboptimal, or inadequate, reflecting the feasibility of reaching a final pathological diagnosis. Moreover, to validate the clinical utility of the coelioscopy, biopsy-derived findings were compared with the histopathology of matched necropsy tissues to assess concordance in morphological findings.

### Statistics

Descriptive statistical analyses (mean ± SD, median, minimum–maximum) of quantitative data for CES and EVS were conducted using GraphPad Prism (v10; GraphPad Software, San Diego, CA, USA).

## RESULTS

Diagnostic coelioscopy and coelioscopic biopsy were successfully performed in all Northern boobooks, with a mean body mass of 192.5 ± 18.1 g (median, 194 g; range, 162–211 g). Minor bleeding occurred during portal entry or tissue sampling; however, it was transient and self-limiting within minutes without additional intervention. No major complications, including intraoperative mortality, cardiopulmonary collapse, or uncontrollable haemorrhage requiring termination of the procedure, were observed.

The CES and EVS are summarised in Table 2. Coelomic entry using a 14-Fr operating sheath and a 2.7-mm telescope was generally rated as easy. In terms of organ visualisation (Figure 2), the lung, proventriculus, ventriculus, liver, kidney, and gonad were consistently well visualised (EVS ≥ 4). The heart, spleen, and intestines showed moderate identifiability (3 ≤ EVS < 4), whereas the cloaca and adrenal gland were difficult to locate (2 ≤ EVS < 3). The oesophagus, gall bladder, pancreas, and ureters were classified as very difficult to identify (EVS < 2). Notably, live nematodes near the air sac membranes were incidentally observed in two individuals; however, attempts to retrieve the parasites were unsuccessful.

**Table 2:** Coelioscopic scores for coelomic entry and organ visibility in eight rescued Northern boobooks (*Ninox japonica*), graded on a 5-point ordinal scale

Items		Mean ± SD	Median	Min	Max
Coelomic entry score^a^		5.00 ± 0.00	5.00	5.00	5.00
Endoscopic visibility scores^b^	heart	3.88 ± 1.25	4.00	1.00	5.00
	lung	4.75 ± 0.46	5.00	4.00	5.00
	oesophagus	1.25 ± 0.71	1.00	1.00	3.00
	proventriculus	4.38 ± 0.74	4.50	3.00	5.00
	ventriculus	4.50 ± 0.76	5.00	3.00	5.00
	liver	4.75 ± 0.46	5.00	4.00	5.00
	gall bladder	1.75 ± 1.39	1.00	1.00	4.00
	spleen	3.75 ± 0.71	4.00	3.00	5.00
	pancreas	1.25 ± 0.71	1.00	1.00	3.00
	small intestine	3.13 ± 1.25	3.00	1.00	5.00
	large intestine	3.00 ± 1.07	3.00	1.00	5.00
	cloaca	2.63 ± 1.19	3.00	1.00	4.00
	kidney	4.00 ± 0.76	4.00	3.00	5.00
	ureter	1.50 ± 0.93	1.00	1.00	3.00
	adrenal gland	2.00 ± 1.51	1.00	1.00	5.00
	gonad	4.13 ± 0.64	4.00	3.00	5.00

^a^Coelomic entry score: 1 = inaccessible (>15 min); 2 = poor (11–15 min); 3 = fair (6–10 min); 4 = good (2–5 min); and 5 = excellent (<2 min); ^b^Endoscopic visibility score: 1 = very poor (barely discernible or not visualised, even after prolonged inspection); 2 = poor (requiring substantial searching and manipulation); 3 = fair (requiring moderate searching and manipulation); 4 = good (readily locatable with slight manipulation); and 5 = excellent (immediate, clear visualisation with little visceral manipulation)

**Figure 2:**
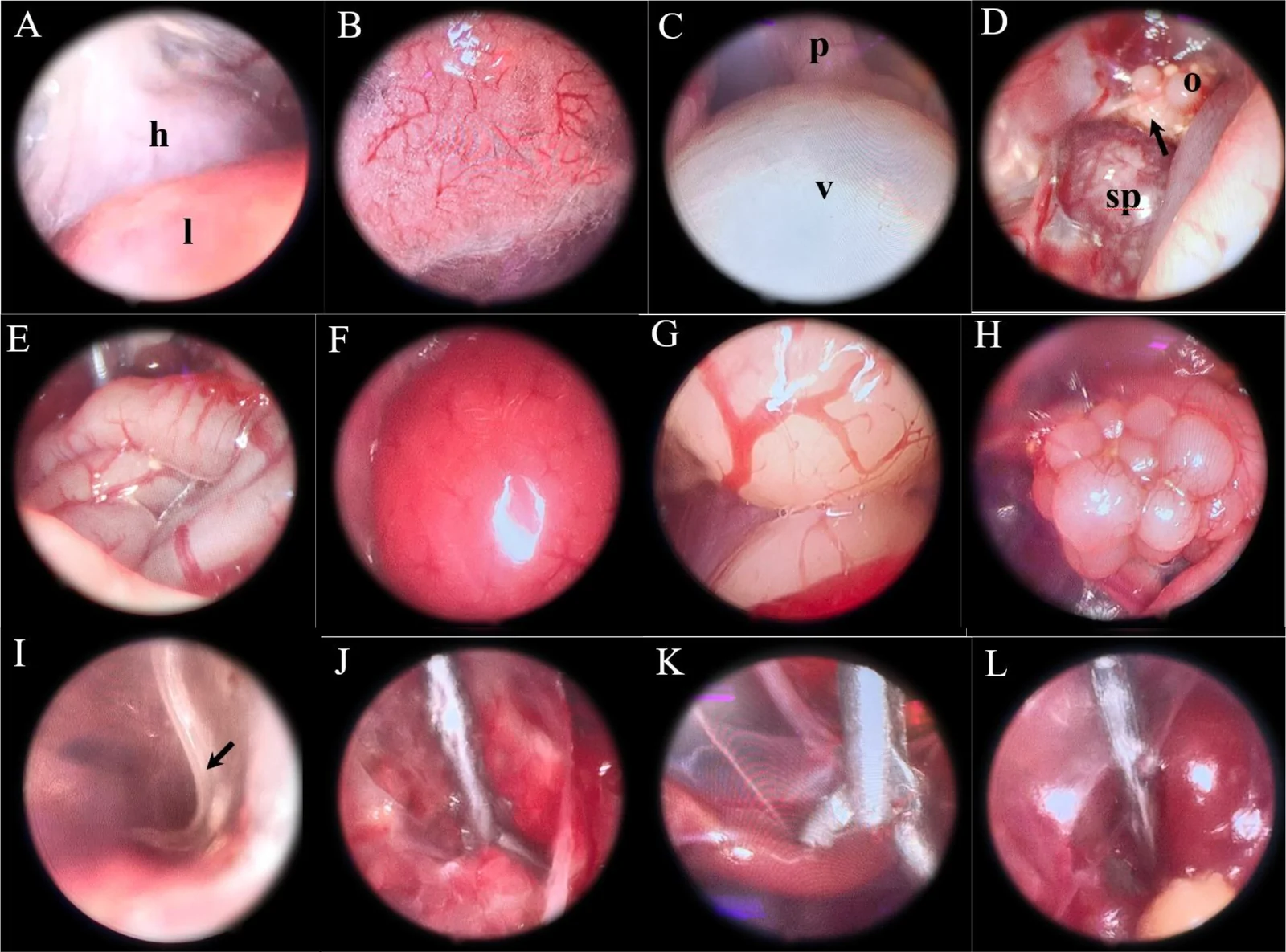
Coelioscopic views of visceral organs and coelioscopy-guided biopsies in Northern boobooks (*Ninox japonica*) (A) Heart (h) and liver (l); (B) lung covered by serosal membrane; (C) proventriculus (p) and ventriculus (v); (D) spleen (sp), immature ovarian follicles (o), and adrenal gland (arrow); (E) loops of the small intestines; (F) cranial division of the kidney; (G) bilateral testes; (H) left ovarian follicles; (I) incidental air sac nematode (arrow); (J) pulmonary biopsy *in situ*; (K) hepatic biopsy *in* *situ*; and (L) renal biopsy *in situ. *Organs not shown were either not photographed or not successfully captured during the procedure

Table 3 summarises the results of confirmation of target organ, diagnostic quality, and diagnostic adequacy for the biopsy specimens. Of the 24 biopsy samples evaluated, two-thirds demonstrated definitive identification of the target organ based on distinct histological features (lung, *n* = 6; liver and kidney, *n* = 5 each), indicating a generally acceptable level of targeting accuracy. However, specimens graded as unidentifiable, where the target tissue was not successfully harvested, occurred across all organ types (liver, *n* = 2; lung and kidney, *n* = 1 each). With respect to diagnostic quality, kidney specimens showed the most consistent tissue preservation, with seven samples graded as good, while liver specimens also demonstrated favourable results, with five samples receiving a good grade. Crush artefact was observed in all organs, ranging from minimal to moderate in severity, although the pattern and extent of tissue damage differed among organs. In particular, lung specimens exhibited more pronounced artefactual changes, including crush injury, atelectasis, desiccation, and fragmentation attributable to specimen handling, compared with other organs. These morphological alterations were reflected in the final assessment of diagnostic adequacy: whereas kidney and liver specimens more frequently achieved an adequate diagnostic rating, lung specimens were predominantly classified as suboptimal (Figure 3).

**Table 3:** Confirmation of target organ, diagnostic quality, crush artefact severity, and diagnostic adequacy of coelioscopic-guided biopsy specimens in Northern boobooks (*Ninox japonica*)

Target organ	Confirmation^a^				Diagnostic quality^b^					Crush artefact^c^				Diagnostic adequacy^d^		
	definitive	probable	unidentifiable		good	limited	poor	missed		minimal	mild	moderate		adequate	suboptimal	inadequate
Lung	6	1	1		4	2	2	0		3	2	3		1	6	1
Liver	5	1	2		5	1	0	2		2	1	3		3	3	2
Kidney	5	2	1		7	0	0	1		3	3	1		4	3	1

^a^Whether the harvested tissue was identifiable as the intended target organ; ^b^Whether representative histological structures required for diagnosis were sufficiently preserved and assessable, with consideration of the severity of crush artefacts; ^c^Samples for which tissue acquisition was unsuccessful (liver, *n* = 2; kidney, *n* = 1) were omitted from the counts of crush artefacts; ^d^Whether the specimen was both representative of the intended target organ and preserved sufficient histological architecture to permit a definitive diagnosis

**Figure 3:**
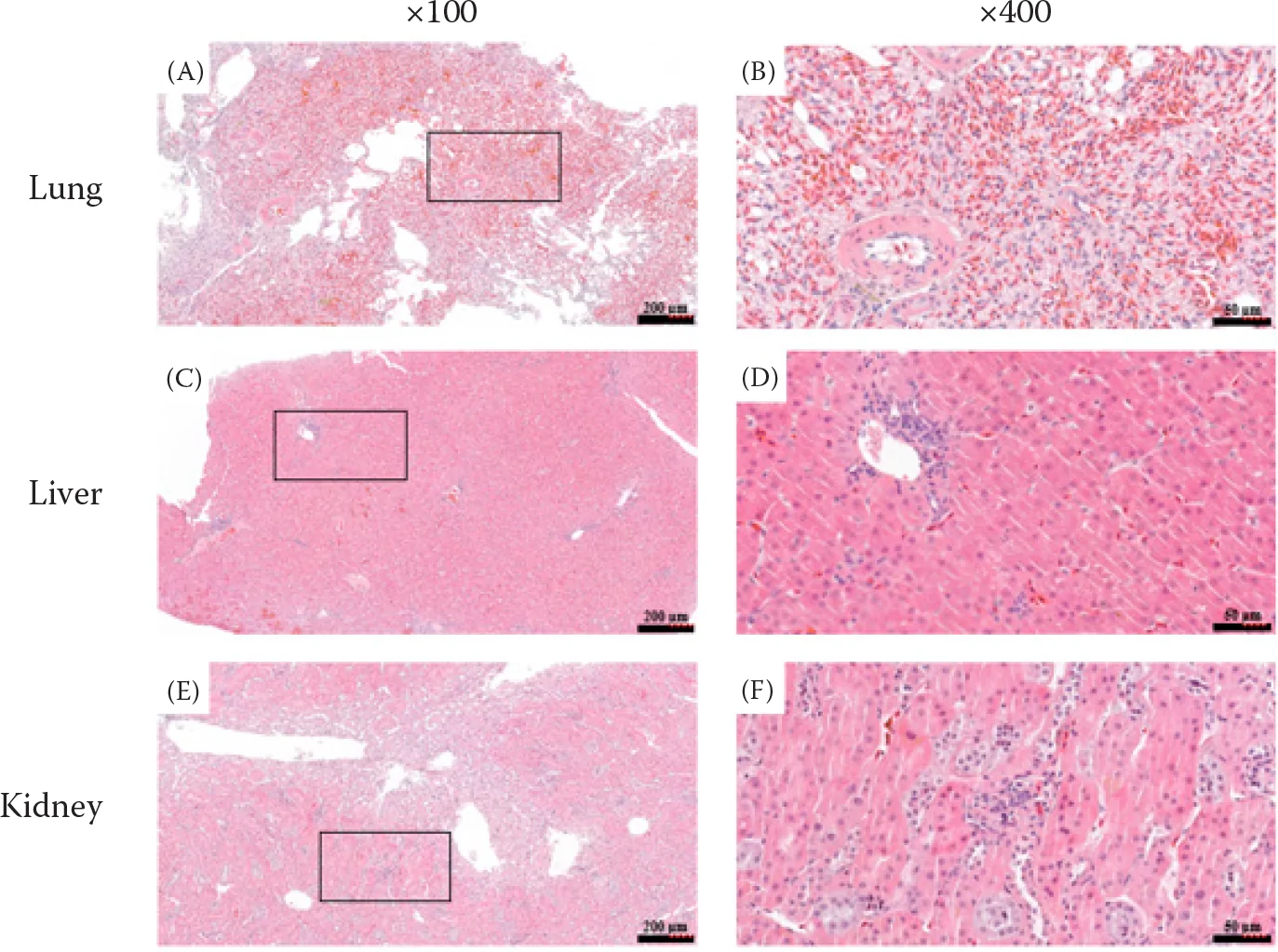
Histological examinations of coelioscopic-guided biopsy specimens from pulmonary (A,B), hepatic (C,D), and renal (E,F) tissues Low-magnification views (×100; A,C,E) show overall tissue architecture, while corresponding higher-magnification images (×400; B,D,F) highlight cellular detail. Pulmonary biopsy samples (A,B) exhibit limited diagnostic quality, characterised by moderate crush artefact and parenchymal distortion, resulting in suboptimal diagnostic adequacy. In contrast, hepatic biopsy sections (C,D) show well-preserved lobular architecture with minimal crush artefact and are considered diagnostically adequate. Renal biopsy specimens (E,F) demonstrate preserved tubular and glomerular structures with minimal crush artefact, supporting good diagnostic quality and adequacy. Haematoxylin and eosin stain; scale bar = 200 μm for (A,C,E) and 50 μm for (B,D,F)

Among the four individuals that underwent necropsy, two biopsy specimens that had failed to yield target tissue (one liver and one kidney) were excluded from the morphological comparison with necropsy findings. Among the comparable samples (Figure 4), two lung and two kidney specimens were recorded as having no lesions on biopsy; however, corresponding necropsy of the same individuals revealed mild, multifocal mononuclear cell infiltration. In the remaining cases, biopsy and necropsy findings were generally concordant. In two cases (one liver and one kidney), no histopathological lesions were identified either in the biopsy specimen or the corresponding necropsy tissue. In contrast, in two liver specimens, biopsy revealed only inflammatory cell infiltration, whereas necropsy additionally demonstrated mild vacuolation.

**Figure 4:**
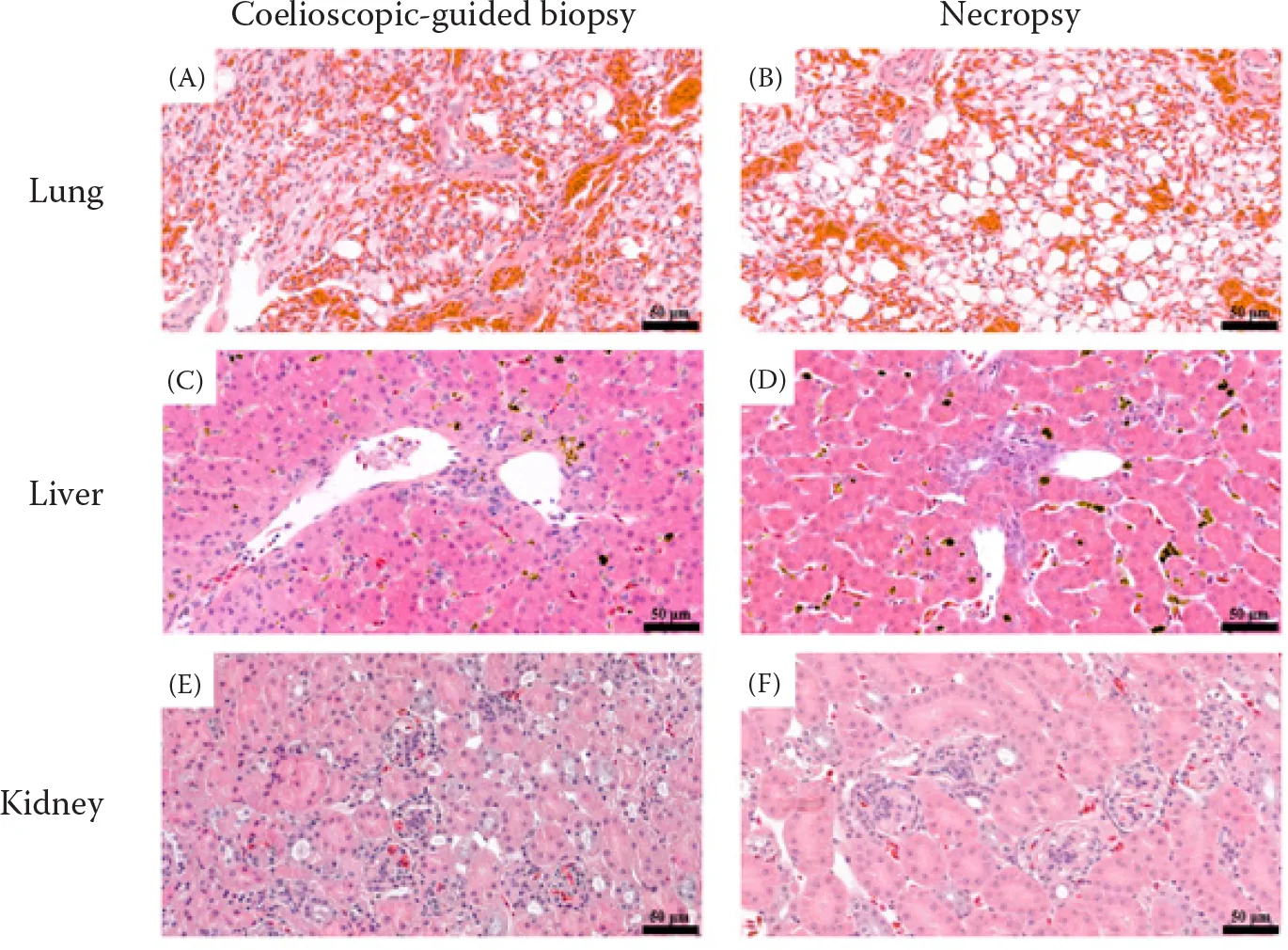
Comparative histological examinations of pulmonary (A,B), hepatic (C,D), and renal (E,F) tissues obtained by coelioscopic-guided biopsy (A,C,E) and corresponding necropsy samples (B,D,F) Pulmonary biopsy sections (A) exhibit variable degrees of parenchymal compression and artefactual distortion compared with the corresponding necropsy tissue (B), in which infundibular architecture is more clearly preserved. Hepatic biopsy samples (C) demonstrate preserved lobular architecture with multifocal mononuclear cell infiltration, comparable to findings observed in necropsy specimens (D). Renal biopsy sections (E) show well-maintained tubular and glomerular structures with mild crush artefact, consistent with the corresponding post-mortem tissue (F). Haematoxylin and eosin stain; magnification ×400; scale bar = 50 μm

## DISCUSSION

In this study, the coelioscopic approach through the left caudal thoracic air sac showed high technical feasibility in Northern boobooks, consistently achieving the highest CES. This favourable outcome is likely attributable to both the anatomical accessibility of the air sac system and the selection of appropriately sized endoscopic equipment. Particularly, the calibre of the rigid telescope is critical for balancing procedural safety with visual quality; thus, it should be tailored to the patient’s body size and the available working space within the coelom (Divers 2010). In routine avian coelioscopy, 2.7 mm rigid telescopes have been most commonly used across a wide range of species, including a chicken (*Gallus gallus*), pigeons (*Columba livia*), cockatiels (*Nymphicus hollandicus*) and multiple raptor taxa (Pye et al. 2001; Muller et al. 2004; Hernandez-Divers et al. 2006; Zaheer et al. 2020). Although a 1.9 mm system may be preferred in very small patients below 100 g (Hernandez-Divers 2005), diagnostic coelioscopy using the 2.7 mm system has been reported even in extremely small birds such as budgerigars (Upadhyay et al. 2023). However, accommodating an oversized instrument requires a larger entry portal, which increases the potential for extensive tearing of air sac membranes. This can, in turn, compromise the airtight seal of the air sac system, thereby exacerbating anaesthetic gas leakage and hypoventilation and predisposing the patient to an unstable plane of anaesthesia (Touzot-Jourde et al. 2005; Seok et al. 2016). The greater bulk of equipment can also constrain manoeuvrability within the confined coelomic space. Overall, given their mean body mass (192.5 ± 18.1 g), a 2.7 mm endoscopic system in Northern boobooks is an appropriate choice that balances procedural safety with diagnostic performance.

In avian coelioscopy, the left-sided approach is the most preferred technique, as it provides broad visual access to the coelom and allows easy inspection of gonads regardless of sex, particularly given the unilateral development of the left ovary in most female aves (Pollock and Orosz 2002). Accordingly, this approach was adopted in this study, and the results in Northern boobooks corroborate these advantages; the gonads and major visceral organs, including the lung, liver, proventriculus, and ventriculus, were highly visible. The high visibility can be explained by their proximity to the entry point and their well-defined morphology. With minor instrumental manipulation, the heart, spleen, and intestinal tract could also be found without significant challenge. This is likely attributable to the anatomical continuity afforded by the left caudal thoracic air sac, which permits extension of exploration into both the cranial thoracic and abdominal air sacs. A notable observation was that, despite the study being conducted during the breeding season of Northern boobooks, no marked impairment in locating target organs was encountered. Gonadal hypertrophy during the reproductive period can obscure the spleen and adjacent viscera (Hernandez-Divers et al. 2006), yet this effect was not evident in this study. This observation may be associated with suppression of breeding activity induced by cumulative stress of the injury and captivity (Fischer and Romero 2019).

Organs with EVS below 3 reflect either an inherent limitation of single-portal access or a size-related challenge. These constraints are interpreted as a consequence of anatomical blind spots arising from visceral topography and differences in the visual field afforded by the selected air sac route. Attempts to locate these structures via prolonged exploration or excessive manipulation may increase the likelihood of iatrogenic damage. Therefore, preliminary diagnostics are warranted to localise the lesion and to inform an approach optimised for the organ of interest.

Indeed, alternative portals can also be considered depending on the region of interest, including right-sided, ventral, and interclavicular approaches. As suggested in previous literature, organ visibility varies depending on the route employed (Divers 2010). For instance, the right-sided approach is suitable for organs that showed limited identification in this study, such as the pancreas. Meanwhile, the interclavicular approach facilitates the evaluation of cranial coelomic structures, and the ventral approach is better suited for assessing bilateral hepatic lobes or patients presenting with coelomic fluid. For this reason, the judicious selection of entry portals, whether single or multiple, tailored to the individual case is essential for visualising otherwise poorly accessible structures. However, because the present study was confined to a single route, future studies comparing organ visibility across approaches are needed to support case-specific portal selection.

Distinct from the above limitations, the low visibility of the ureter reflects a size-related issue. In birds, the ureter can be identified along the ventromedial surface of the kidney (Divers 2010); however, in the small-bodied Northern boobook, the structure was so fine that it was difficult to clearly distinguish under coelioscopic view. Signs such as ureteral distension or uric acid deposition would have facilitated its identification, but no such changes were evident in the present study.

The varying degrees of crush artefacts across all organ specimens in the present study mirror findings in cinereous vultures (*Aegypius monachus*), where a double-portal entry using larger 15-Fr biopsy forceps was employed (Seok et al. 2016). Although the size of forceps and portal configuration differed from those used in the Northern boobooks, the persistence of artefacts in both investigations is noteworthy. This implies that the artefact formation was caused by mechanical disruption inherent to the grasping action of the biopsy forceps, rather than by differences in instrument diameter or portal configuration. Nevertheless, similar to findings in cinereous vultures that central parenchyma remains amenable for histology despite peripheral damage (Seok et al. 2016), hepatic and renal samples in the present study demonstrated high diagnostic quality and concordance with necropsy findings. This supports coelioscopic biopsy as a reliable modality for hepatorenal assessment, provided that adequate tissue targeting is achieved.

Another consideration relates to the absence of pre-incision of the serosal membrane. Although a previous study recommends serosal pre-incision using endoscopic scissors for both pulmonary and hepatic sampling (Divers 2010), this step was not performed in the present study. The potential benefits of this step were only recognised post-hoc, and its omission may have contributed to the failure to obtain samples from two liver specimens. Despite this limitation, the remaining liver samples generally maintained high diagnostic quality, likely because sampling targeted the hepatic margin, where forceps-based tissue acquisition is anatomically more feasible. In contrast, the avian lung is a rigid organ firmly affixed to the thoracic wall (Maina 2015). When pulmonary tissue is sampled without serosal pre-incision, surface compression and traction are unavoidable, resulting in substantial shear and torsional forces as the tissue passes through the intact serosa. These mechanical stresses may account for the pronounced artefacts observed in lung specimens, which directly corresponded to their low diagnostic adequacy. Accordingly, while the high concordance of liver and kidney biopsies supports their reliability for hepatorenal assessment, pulmonary evaluation appears to require procedural refinement, such as serosal pre-incision, to reduce tissue damage and improve specimen representativeness. Furthermore, partial discrepancies between biopsy and necropsy findings underscore the risk of false-negative results in cases of non-diffuse pathology, as limited tissue sampling may not include representative diseased areas (Saggese et al. 2010). Coelioscopic biopsy findings should therefore be interpreted as site-specific information and integrated with the overall clinical context.

Several limitations of this study need to be acknowledged. First, given that coelioscopy is an operator-dependent modality, the possibility that the EVS were influenced by the operator’s proficiency cannot be excluded. The technical challenges were also exemplified by non-diagnostic biopsy specimens and unsuccessful parasite retrieval. Furthermore, the study did not fully account for organ-specific anatomical features, such as the serosal membrane, leading to crush artefacts or tissue acquisition failures. These findings underscore that an approach optimised for each organ must be considered beforehand when targeting visceral lesions via coelioscopy.

Second, the study population comprised a limited number of rescue cases with poor prognoses scheduled for euthanasia. Although no overt confounding lesions were identified in the present cases, such birds may harbour co-morbidities including coelomic adhesions, effusion, or organomegaly, which may have adversely affected visibility scores compared with healthy birds. In particular, the immediate euthanasia following the procedure precluded any evaluation of postoperative recovery and procedural safety, representing a key limitation of this study.

Third, histopathological comparison was restricted to half of the birds, confining the biopsy–necropsy comparison to a subset of the cohort. Nevertheless, this cohort reflects the clinical reality of wildlife rescue centres, conferring practical relevance. Larger cohorts of surviving patients are warranted to assess long-term outcomes, complications, and concordance between coelioscopic and histopathological findings.

In summary, diagnostic coelioscopy with biopsies constitutes a feasible modality for evaluating coelomic pathologies in Northern boobooks. By facilitating direct visceral inspection, this technique effectively circumvents the diagnostic limitations inherent to wildlife practice. However, because visceral identifiability and the diagnostic quality of biopsy specimens depend on portal selection and anatomical constraints, a meticulous surgical strategy is imperative. Consequently, clinicians must optimise the protocols relative to the target pathology to mitigate the limitations of single-portal access and ensure diagnostic accuracy.
